# Electro-optic switching in metamaterial by liquid crystal

**DOI:** 10.1186/s40580-015-0054-6

**Published:** 2015-12-01

**Authors:** Yeon Ui Lee, Junghee Kim, Jeong Weon Wu

**Affiliations:** Department of Physics, Quantum Metamaterials Research Center, Ewha Womans University, Seoul, 120-750 Korea

**Keywords:** Metamaterials, Electro-optics, Liquid crystal

## Abstract

Electro-optic switching of reflection and refraction is experimentally demonstrated in metasurface liquid crystal cell. Negative metasurface is fabricated by focused-ion-beam milling, and twisted nematic cells are constructed with complementary double-split ring resonator and V-shape slot antenna metasurface. By application of an external voltage, electro-optic switchings are achieved in reflection and refraction. It has a strong implication for applications in spatial light modulation and wavelength division multiplexer/demultiplexer in a near-IR spectral range.

## Introduction

Metamaterial is artificially structured material with subwavelength-sized inclusions, which exhibits responses not readily observed in natural material. While 3D metamaterial is rather difficult to fabricate, 2D metamaterial can be fabricated on a substrate by well-established processes of photo-lithography, electron beam lithography (EBL), and focused ion beam (FIB) milling.

2D metamaterial received a wide attention as an extension of frequency selective surface, leading to a class of metamaterial called metasurface. Metasurface has important application such as flat optical elements replacing bulky glass optical elements as well as extraordinary refraction satisfying generalized Snell’s law. In order to achieve a functionality in metasurface, metasurface can be combined with liquid crystal to construct a metasurface liquid crystal (MSLC) cell, allowing an electro-optic control of the designed optical response of metasurface.

In this work we introduce two examples of electro-optic control of near-IR optical response in MSLC cell. The first example makes use of reflection resonances in metasurface with inclusion of split-ring resonators, and the alignment of twisted nematics in MSLC cell is controlled electro-optically permitting a functionality of tunable reflection. As the second example, we adopt a phase-discontinuity metasurface with inclusion of V-shape antenna array, which exhibits broadband extraordinary refraction in a cross-polarization scattered light. MSLC cell is built in a way similar to the first example, and a functionality of tunable refraction is achieved.

After a discussion on Babinet principle to compare scattering fields from dipole and slot antennas constituting metasurface, a fabrication of metasurface and construction of MSLC cell are introduced. The near-IR optical characteristics is presented, and the electro-optic controls are demonstrated.

## Review

### Positive/negative metasurfaces and Babinet principle

For near-IR wavelength applications, metasurface possessing hundreds nanometer-sized inclusions can be fabricated by either EBL or FIB milling [1]. In order to obtain metamaterials with optical response in near-IR spectral range, it is necessary to prepare nano-sized meta-particle as the basic subwavelength-sized inclusion unit, which is nominally fabricated through direct patterning such as EBL and FIB milling [2]. EBL involves a multi-step process including nano-sized pattern transfer to polymer resist by e-beam exposure, subsequent metal deposition, and final lift-off yielding a positive meta-structure with resonance appearing in transmission spectra. On the other hand, FIB milling is a one-step process of producing nano-sized apertures and is a major tool to fabricate near-IR optical elements in a negative meta-structure pattern [3, 4]. As far as pattern transfer is concerned, details of fabrication processes are in contrast, since beam of electrons exposes polymer resist in EBL with metallic film intact, while focused-ion beam in FIB milling makes an ion bombardment on metallic film directly, which yields different pattern edge roughness and substantial distortion in linewidth of nano-sized patterns.

When an electro-optic control of optical properties is studied in MSLC cell, negative metasurface fabricated by FIB milling has an advantage over positive metasurface, since a large metallic area of a negative metasurface can be employed as electric contact needed for electro-optic control in MSLC cell. Therefore, in both examples of reflection and refraction control by electro-optical means, we fabricated negative metasurfaces by FIB milling.

Optical responses such as reflection and refraction from metasurface are determined by scattering characteristics of electromagnetic fields from subwavelength-sized antennas constituting metasurface. Noting that positive and negative metasurfaces are composed of dipole and slot antennas, respectively, they are complementary when the same pattern is employed in fabrication via EBL for positive metasurface and FIB milling for negative metasurface.

**Fig. 1:**
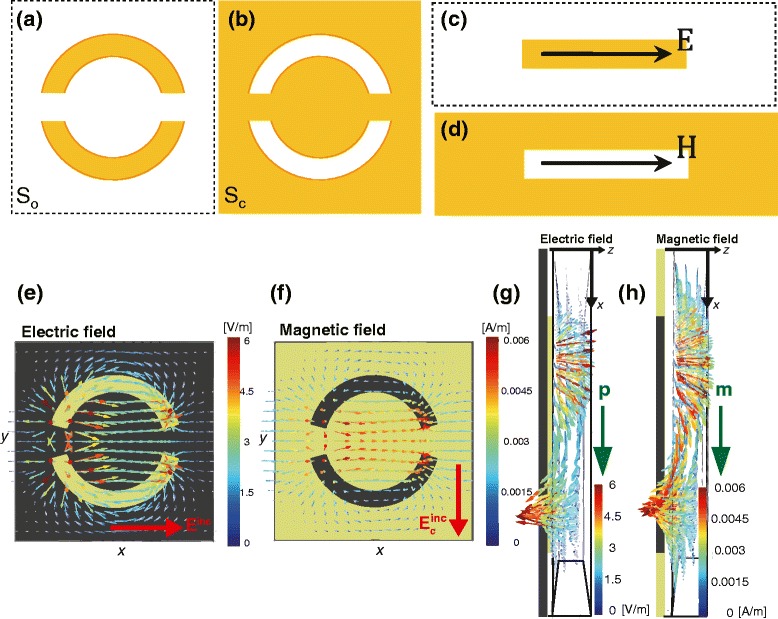
Schematics of complementary diffraction screens are shown. (**a**) The original screen with metallic double-split ring resonators and (**b**) its complementary screen with double-split ring apertures are shown. (**c**) and (**d**) are dipole and slot antennas with complementary electric and magnetic fields, respectively. Both electric and magnetic fields are along the horizontal axis. Simulation results of the DSRR electric vector fields are shown in (**e**) and (**g**), and cDSRR magnetic vector field are shown in (**f**) and (**h**). The red arrows represent polarization direction of incident wave in DSRR and cDSRR, respectively. The green arrows represent induced dipoles in DSRR and cDSRR, respectively

Babinet principle is a guideline to design subwavelength inclusions, where the reflection spectrum of a negative metasurface can be predicted based on the known transmission spectrum of a positive metasurface by employing the complementary screen [5, 6]. Figure 1(a) and (b) show schematics of two complementary screens. Original diffraction screen with area *S*_*o*_ and its complementary screen with area *S*_*c*_ constitutes the entire surface area *S* as *S*_*o*_+*S*_*c*_=*S*.

Relation of diffraction fields between the two complementary screens such as those shown in Fig. 1(c) and (d) is described by Babinet principle [7, 8]. Let the incident fields at the original screen *S*_*o*_ and its complementary screen *S*_*c*_ be the original field $\vec {E}^{0}$ and $\vec {H}^{0}$ and the complementary field $\vec {E}^{0}_{c}$ and $\vec {H}^{0}_{c}$ defined as below.

$$\begin{array}{@{}rcl@{}} \begin{array}{rll} \textrm{Original screen~ S\(_{o}\):} & \vec{E}^{0} &,~\vec{H}^{0} \\ \textrm{Complementary screen~ S\(_{c}\):} & \vec{E}^{0}_{c}\!=-\!\sqrt{\frac{\mu_{0}}{\epsilon_{0}}}\vec{H}^{0} &,\!~\vec{H}^{0}_{c}\,=\,\sqrt{\frac{\epsilon_{0}}{\mu_{0}}}\vec{E}^{0} \end{array} \end{array} $$

Note that *S*_*o*_ and incident field are replaced by *S*_*c*_ and 90°-rotated incident field. For an infinitely thin conducting screen, symmetry properties and boundary conditions lead to Babinet principle, which states that transmission spectrum of *S*_*o*_ is identical to reflection spectrum of *S*_*c*_ for two complementary incident fields. Babinet principle can be applied to the radiation patterns of dipole and slot antennas as illustrated in Fig. 1(c) and (d). When the slot antenna is illuminated by a magnetic field along the slot, the radiation pattern is the same as that of the dipole antenna illuminated by an electric field along the rod.

Simulation results of double-split ring resonator (DSRR) electric vector fields are shown in Fig. 1(e) and (g), and complementary double-split ring resonator (cDSRR) magnetic vector field are shown in Fig. 1(f) and (h) when illuminated by the incident electromagnetic plane wave from *z*<0. The red arrows represent polarization direction of incident wave in DSRR and cDSRR, respectively. The green arrows in Fig. 1(g) and (h) represent induced dipoles in DSRR and cDSRR, respectively.

**Fig. 2:**
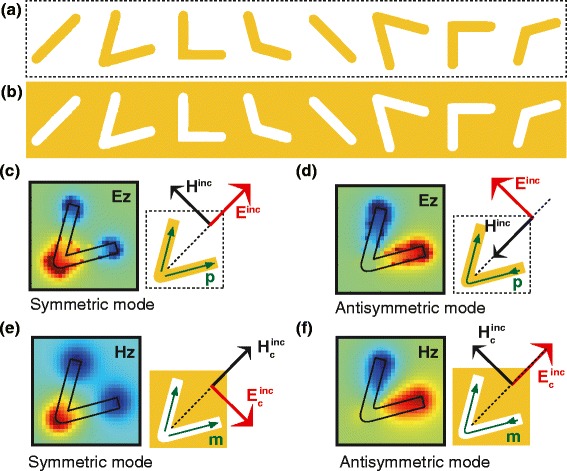
Schematics of complementary diffraction screens are shown. (**a**) The original screen with metallic arrays of V-shaped dipole and (**b**) its complementary screen with V-shaped slot antennas are shown. Schematics of the first-order excited (**c**) symmetric and (**d**) antisymmetric electric modes in a V-shaped dipole antenna are shown. Schematics of the first-order excited (**e**) symmetric and (**f**) antisymmetric magnetic modes in a complementary V-shaped dipole antenna for an incident electric field polarized either perpendicular to the antenna symmetry axis or along the antenna symmetry axis are shown

The other example of complementary metasurfaces of V-shaped antennas are shown in Fig. 2(a) and (b). In an array of V-shaped antennas, the folding angle of V-shape is designed such that phase delay of cross-polarization scattered light varies from 1st to 8th antennas in the amount of 2 *π* to achieve an extraordinary refraction. The first 4 antennas possess mirror symmetry with respect to 45° straight line, while the next 4 antennas possess mirror symmetry with respect to 135° straight line. Owing to the mirror symmetry, the scattering pattern of cross-polarization scattered light are identical for arrays of V-shaped dipole and slot antennas when the incident electric field is horizontally polarized [9].

Figure 2(c) and (d) correspond to symmetric and antisymmetric electric modes in a V-shaped dipole antenna, respectively, while Fig. 2(e) and (f) correspond to symmetric and antisymmetric electric modes in a V-shaped slot antenna, respectively.

### Transmission and reflection spectra and fabrication of metasurface

**Fig. 3:**
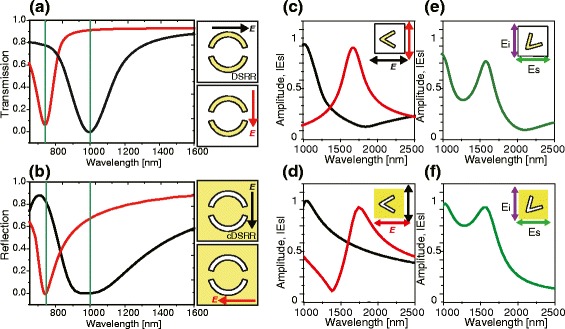
Positive and negative metasurfaces with their (**a**) transmission and (**b**) reflection spectra are shown. Amplitudes of scattering field, *E*_*s*_, from positive and negative metasurfaces are shown in (**c**) and (**d**). Amplitudes of cross-polarization scattered field amplitudes from positive and negative metasurfaces are shown in (**e**) and (**f**)

Once the scattering fields from dipole and slot antennas constituting metasurfaces are identified, we calculate transmission and reflection spectra. Figure 3(a) and (b) correspond to transmission and reflection spectra of positive and negative metasurfaces of DSRR, respectively. The resonance at 750 nm for electric field along vertical axis in DSRR corresponds to the resonance at 750 nm for electric field along horizontal axis in cDSRR as shown in red curves in Fig. 3(a) and (b). The resonance at 1000 nm for electric field along horizontal axis in DSRR corresponds to the resonance at 1000 nm for electric field along vertical axis in cDSRR as shown in black curves in Fig. 3(a) and (b). When compared with the resonance at 750 nm we find that the resonance at 1000 nm shows a much broaderband width in cDSRR than in DSRR, which can be attributed to a higher Joule heating loss in complementary negative metasurface.

In Fig. 3(c) and (d) are shown the amplitudes of scattered electric field in V-shaped dipole and slot antennas constituting metasurface for vertical and horizontal polarized incident lights. Black and red curves correspond to symmetric and antisymmetric modes, respectively. Spectra of scattered field amplitude in symmetric mode shows a complementary feature as described by Babinet principle. On the other hand, antisymmetric mode exhibits a feature somewhat deviating from Babinet’s principle, which can also be attributed to a higher Joule heating loss in complementary negative metasurface.

Figure 3(e) and (f) show cross-polarization scattered field amplitudes in dipole and slot V-shaped antenna with folding angle 45°. V-shaped antenna is oriented with the symmetric axis along 45° with respect to horizontal axis, and the green curves correspond to horizontal polarization scattered field amplitude for vertical polarized incident light. We find the spectral features of Fig. 3(e) and (f) follow Babinet principle.

**Fig. 4:**
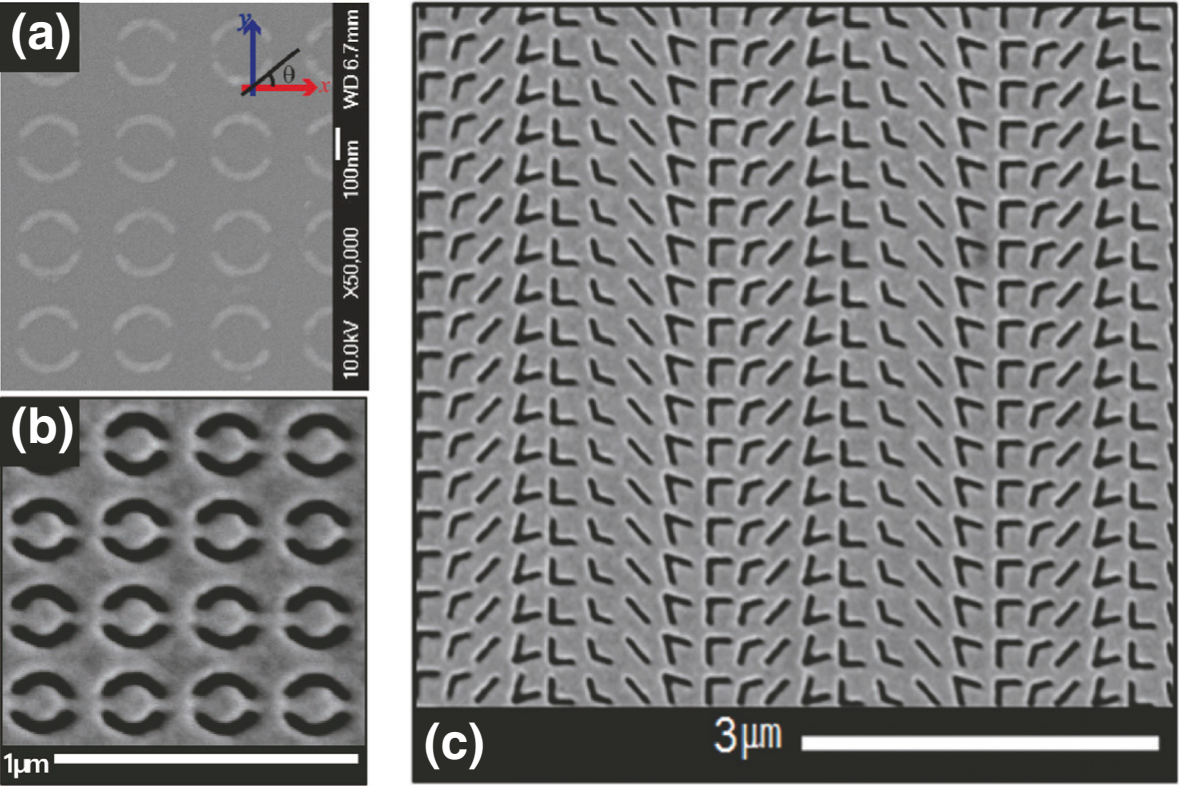
Scanning electron microscopy images of (**a**) positive, (**b**) negative double-split ring resonators and (**c**) negative V-shaped slot antennas are shown

Negative metasurfaces are fabricated by FIB milling. Once 30nm-thick Au film is e-beam evaporated on a 1mm-thick fused quartz substrate deposited with 3nm-thick Ti adhesion layer, FIB milling is performed. With the accelerating voltage fixed at 30 *kV*, nano-sized slot antenna array of double-split ring resonators was successfully milled at the ion beam current 1.5 *pA* with milling depth setting of 40 nm as can be seen in the SEM image of Fig. 4(b) and (c).

Arrays of DSRR are fabricated by EBL and FIB milling as shown Fig. 4(a) and (b), respectively, which are complementary metasurfaces.

In an FIB milling, Au layers are remnant inside the arc-patterned nano-apertures, namely, the dielectric fused quartz substrate is not fully shaped as an arc pattern, which results in broadened reflective resonances of nano-sized DSRR apertures. With the same process parameters a linear array of eight V-shape apertures with the lattice constant *Γ* of 2400 *n* *m* is fabricated by FIB milling, the SEM image of which is shown in Fig. 4(c). V-shaped slot antenna is designed to provide a phase discontinuity of ${{d\Phi } \over {dx}}= -{{2\pi }\over \Gamma }=-2.618 rad/\mu m $ at complementary V-shaped metasurface (cVMS).

### Construction of MSLC cell

By adopting a twisted nematic liquid crystal (TNLC) cell structure with metasurface as one of windows of TNLC cell, electro-optic control of transmission or reflection resonances of metamaterial can be achieved for a normally incident light [10–12]. A large area of metal surface in negative metasurface can serve as an electrode keeping each slot antenna spatially separated from others. The spatial separation of slot antennas is a key feature in keeping the characteristic meta-resonances of metasurface, which cannot be configured in positive metasurface when an electro-optic application is sought in MSLC cell [11].

We prepared a liquid crystal cell by sandwiching a rubbed polyimide(SE-5291, Nissan Chemical Industries, Ltd.)-coated ITO glass and a rubbed poly-vinyl-alcohol(PVA, *M*_*w*_≈ 205K, Aldrich)-coated fused quartz substrate deposited with negative meatsurface. By capillary-filling the cell of 12 *μ*m cell gap with nemtaics, we obtained an MSLC cell with the liquid crystal in twisted nematic configuration. Nematic liquid crystal is ZLI-2293 (Merck) with *n*_*e*_=1.6313 and *n*_*o*_=1.4990 at 589.3nm, *ε*_∥_ = 14.0 and *ε*_⊥_ = 4.1 at 1.0kHz, possessing the clearing point 85 °C.

**Fig. 5:**
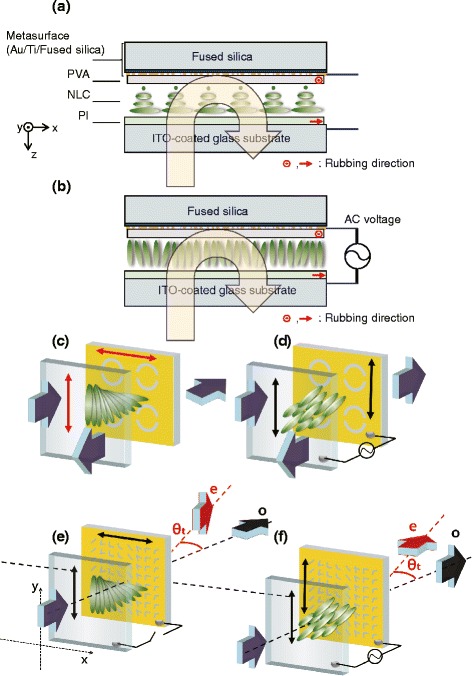
Schematics of cell construction of metasurface liquid crystal is shown. (**a**) in the absence and (**b**) in the presence of of external electric field, the alignments of nematics are different. Schematics of a reflective metamaterial twisted mematics cell are shown, when (**c**) no external voltage is applied and (**d**) an AC voltage is applied. Vertical polarization of an incident beam experiences different reflective resonances upon reflecting from the metamaterial bottom alignment layer [14]. Schematics of a cVMS-TNLC cell is shown with a *y*-polarized incident light, when (**e**) no external voltage is applied and (**f**) an ac voltage is applied. Linear polarization states are orthogonal for ordinary and extraordinary refractions [9]

Figure 5 shows schematics of cell construction of metasurface liquid crystal. In the absence of an external electric field, the alignment of twisted nematic is maintained as shown in Fig. 5(a), and in the presence an external electric field, the alignment of twisted nematic is broken to become isotropic with respect to a normally incident light as shown in Fig. 5(b).

Before we proceed further, we study how the polarization state changes as the beam propagates in a TNLC cell. As a linearly polarized beam propagates through TNLC cell before entering metasurface, we keep track of changes in the polarization state by employing the Jones matrix [9, 13].

$${} {\fontsize{8.8pt}{9.6pt}\selectfont{\begin{aligned} M_{\text{TNLC}}\!=\left(\begin{array}{cc} \cos{\Psi} & -\sin{\Psi} \\ \sin{\Psi} & \cos{\Psi} \end{array} \right)\! \left(\begin{array}{cc} \!\!\cos{X}-i\frac{\Delta}{2}\frac{\sin{X}}{{X}} & \Psi\frac{\sin{X}}{{X}} \\ -\Psi\frac{\sin{{X}}}{{X}} & \!\cos{{X}}+i\frac{\Delta}{2}\frac{\sin{{X}}}{{X}} \end{array} \right) \end{aligned}}}  $$

where

$$\begin{array}{@{}rcl@{}} X=\sqrt{\Psi^{2}+{\left(\frac{\Delta}{2} \right)}^{2}} \end{array} $$

*Ψ* is the total twist angle of the LC director, and the external voltage-dependent phase retardation is *Δ*(*V*)=2*π*{*n*_*e*_(*V*)−*n*_*o*_}*d*/*λ* with *d* the cell thickness.

We express the input and output polarization states as follows, as determined by the orientation angles of the polarizer transmission axes.

$$\begin{array}{@{}rcl@{}} \left(\begin{array}{cc} V_{x} \\ V_{y}\end{array}\right)=\left(\begin{array}{cc}\cos{\phi_{\text{ent}}} \\ \sin{\phi_{\text{ent}}}\end{array}\right),~~ \left(\begin{array}{cc} V'_{x} \\ V'_{y}\end{array}\right)=\left(\begin{array}{cc}\cos{\phi_{\text{exit}}} \\ \sin{\phi_{\text{exit}}}\end{array}\right). \end{array} $$

The transmission intensity through metasurface liquid crystal cell is

$$\begin{array}{@{}rcl@{}} T=|V'^{\ast}\cdot M_{\text{Meatsurface}}M_{\text{TNLC}}V|^{2} \end{array} $$

where *M*_Meatsurface_ is the Jones matrix of metasurface corresponding to Fig. 4(b) and (c).

Figure 5(c) and (d) show how reflection resonance switching is achieved in a reflective cDSRR-TNLC cell, while Fig. 5(e) and (f) show how broadband refraction switching is achieved in a cVMS-TNLC cell [9, 14].

### Electro-optic switching of optical responses in MSLC cell

Mesogene of nematics is highly birefringent as well as polarizable without possessing a permanent dipole moment. In twisted nematic configuration, the electric field of an incident light follows the twisted direction since twisted nematic works as a waveguide. In the presence of an external driving voltage, the nematics in twisted nematic configuration aligns in parallel to the electric field, destroying the twist. We note that an AC driving voltage is applied across metasurface liquid crystal cell since a build-up of any ionic impurities inside nematic liquid crystal can be prevented by an AC driving. Also we note that the interaction of induced dipole moment and external electric field is independent of the sign of external electric field.

The aligned configuration exhibits positive or negative uniaxial property, depending on the difference between ordinary and extraordinary refractive indices. When an optical light propagates along the uniaxial direction, the aligned configuration is optically isotropic. Electro-optic switching in both reflection and refraction makes use of this behavior of twisted nematics.

**Fig. 6:**
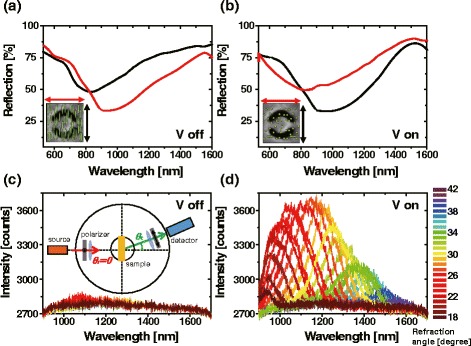
Reflection spectra of metasurface liquid crystal cell is shown for negative metasurface possessing reflection resonances. (**a**) In the absence of an external electric field two reflection spectra are shown for horizontal (red curve) and vertical (black curve) polarized incident lights. (**b**) In the presence of an external electric field two reflection spectra are shown for horizontal (red curve) and vertical (black curve) polarized incident lights. Transmission spectra of extra-ordinary refraction beam from metasurface liquid crystal cell is shown for negative metasurface possessing phase-discontinuity V-shaped slot antennas. Color code refers to the measurement angles. Off and on of spectra can be seen (**c**) in the absence and (**d**) in the presence of an external electric field

Figure 6(a) and (b) show the electro-optic switching of reflection in negative metasurface of Fig. 4(b). In the absence of an external electric field two reflection spectra are shown for horizontal (red curve) and vertical (black curve) polarized incident lights as seen in Fig. 6(a). In the presence of an external electric field two reflection spectra are shown for horizontal (red curve) and vertical (black curve) polarized incident lights in Fig. 6(b). When Fig. 6(a) and (b) are compared, we find that the twisted nematic and aligned configurations leads to the almost identical reflection spectra with the polarization direction swapped each other. The modulation depth depends on the wavelenght and the maximum modulation depth of ≈0.5dB is achieved near resonance for both horizontal and vertical polarized incident lights. Furthermore, the rather broad width of resonance allows the switching operation over 500nm spectral range.

Figure 6(c) and (d) show the electro-optic switching of transmission spectra of extraordinary refraction beam from MSLC cell is shown for negative metasurface possessing phase-discontinuity V-shaped slot antennas. sIn the absence of an external electric field there in no transmission at the measurement angles as seen in Fig. 6(c), and in the presence of an external electric field a transmission is observed at the measurement angles for light with different wavelengths as seen in Fig. 4(d). According to the generalized Snell’s law the angles of reflection *θ*_*r*_ and refraction *θ*_*t*_ are related to the incidence angle *θ*_*i*_ by the generalized Snell’s law, $\sin \theta _{i}+{{1} \over {k_{0}}}{{d\Phi } \over {dx}}=\sin \theta _{t}=\sin \theta _{r}$ [15]. The refraction angle is dependent on the wavelength for a normal incident light, which is obtained from *θ*_*t*_= *θ*_*r*_= arcsin(sin*θ*_*i*_−2*π*/*k*_0_ *Γ*).

## Conclusions

In summary, we experimentally demonstrated an electro-optic switching in metasurface liquid crystal cell. Negative metasurface is fabricated by focused-ion-beam milling, and twisted nematic cells are constructed with complementary double-split ring resonator and V-shape slot antenna metasurface. By application of an external voltage, electro-optic switchings are achieved in reflection and refraction for two different metasurfaces. Switching operation in metasurface liquid crystal cell provides a new photonic device architecture where spatial light modulation and wave division multiplexer/demultiplexer can be achieved in a broadband near-IR spectral range.
